# Pilot-Scale Carbon Capture in a Heat-Pipe-Intercooled Rotating Packed Bed

**DOI:** 10.1021/acs.iecr.4c01614

**Published:** 2025-01-23

**Authors:** James R. Hendry, Jonathan G.M. Lee

**Affiliations:** School of Engineering, Merz Court, Newcastle University, Newcastle upon Tyne NE1 7RU, U.K.

## Abstract

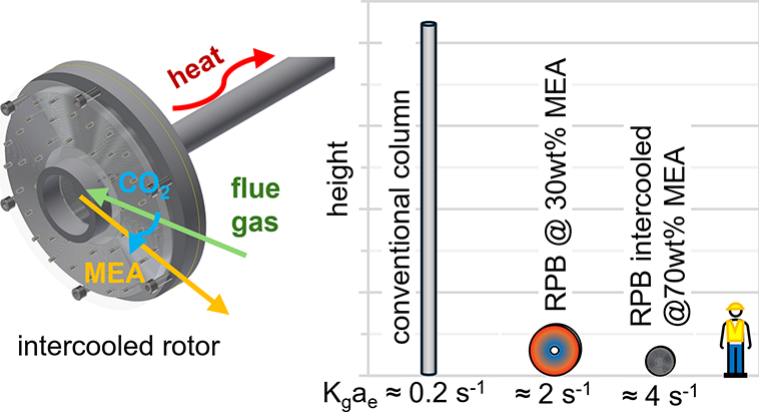

The equipment size and energy penalties of carbon-capture processes can be reduced substantially by using rotating packed beds (RPB) and high-concentration amines. However, intercooling the absorption process is necessary to remove the heat-of-reaction that would otherwise halt CO_2_ absorption in full-scale processes. This paper presents pilot-scale experimental results in carbon capture, using a novel intercooled RPB rotor design that incorporates thermosyphon heat pipes and a variable-area packing. Tests outline the performance benefits of the design and present a correlation for the effects of rotation speed and liquid flow on overall gas-side mass-transfer coefficient (K_g_a_e_). The results show that K_g_a_e_ is improved by 130% in comparison to previous conventional RPB rotor designs, providing an experimental demonstration of the benefits of intercooled RPBs in intensified carbon-capture processes.

## 1 Introduction

Many existing industrial sites have very limited additional space or plant land footprint, and such sites will struggle to retrofit the carbon-capture plants necessary to decarbonize industry. A process intensification^1,2^ approach to designing carbon-capture plants can reduce the equipment size and space requirements. Rotating packed beds (RPBs) can replace the absorption and desorption columns in amine-based carbon-capture processes. This can reduce their size by an order-of-magnitude. Additionally, intercooling the absorption process allows for higher rich amine loadings and energy savings during desorption.^1^ This is of particular interest in intensified carbon-capture processes using RPBs,^3^ where intercooling permits the use of high-concentration amines at practical liquid-to-gas flow ratios (*L*/*G*). This work reports pilot-scale results using a heat-pipe-intercooled rotating packed bed prototype unit, serving as an experimental verification of these benefits.

### 1.1 Rotating Packed Bed for Carbon Capture

RPBs can replace conventional columns in gas–liquid contact processes. Conventional columns rely on gravity and buoyancy forces under Earth’s gravity to achieve contact between gas and liquid phases limited to 1 *N*_g_. Rotating packed beds in carbon-capture applications typically use rotation forces of about 50 *N*_g_. The high-shear environment creates thin well-mixed films of liquid on the RPB packing surface that improve mass-transfer rates. This means smaller equipment sizes at equivalent throughput. Industrial pilots have achieved 90% CO_2_ capture at a 1 ton/day CO_2_ scale. These have demonstrated that this technology is able to achieve an order-of-magnitude size reduction when compared to conventional equipment.^4^

In the UK, government track-1 cluster projects plan to install 5 Mtpa of CO_2_ capture capacity by 2030.^5^ Eighty percent of the UK’s industrial emitters are small-scale (<300 tCO_2_/day).^6^ Costs of carbon capture are dominated by CAPEX at this scale. A niche exists for prefabricated, containerized RPB carbon-capture plants, capable of delivering a 10× size reduction, and 50% cost reduction compared to conventional technologies.^7^ Further size reductions to the RPB technology could be achieved through the use of high-concentration amines.

### 1.2 High-Concentration Amines

As part of a design philosophy based on process intensification, solution concentrations can also be increased to improve the performance of processes and reduce equipment size. Jassim et al.,^8^ Ma and Chen,^9^ and Kolawole^10^ reported experimental investigations using high-concentration amines in RPBs. Viscosity limits amine (monoethanolamine) concentrations to ca. 30 wt % in conventional columns, but RPBs can accommodate 70 wt %: a 4–5× increase in viscosity.^11^ RPBs are less sensitive to the detrimental effects of high viscosities on mass transfer in comparison to conventional columns.^12^ The increased concentration increases the reaction rate and therefore overall mass-transfer coefficient (K_g_a_e_), leading to a further halving in equipment size for the RPB rotor. Reduced solvent recirculation rates also mean that the size of the entire carbon-capture plant is reduced by a similar factor. The regeneration energy supplied to the reboiler is also reduced by ca. 1 GJ/tCO_2_.^3,13^ A smaller rotor also reduces energy use in motor power and gas pressure drop for the RPB. Increasing the solution concentration will also increase corrosion rates, roughly proportionally.^14^ However, a reduced equipment size from intensification makes stainless steel construction cheaper and the consequences of corrosion less impactful. MEA is used in this work as a benchmarking solvent. A full-scale plant is more likely to use a commercial amine blend that are typically less corrosive.^15^ The benefits of a process intensification approach also apply to commercial amine blends. However, the reaction exotherm in high-concentration amines, at practical liquid/gas flow ratios, creates a temperature rise in the amine, which will increase the equilibrium partial pressure of CO_2_ in amine, stopping the absorption process before the target 90% CO_2_ capture rate is reached.

### 1.3 Intercooled Rotating Packed Beds

**Figure 1:**
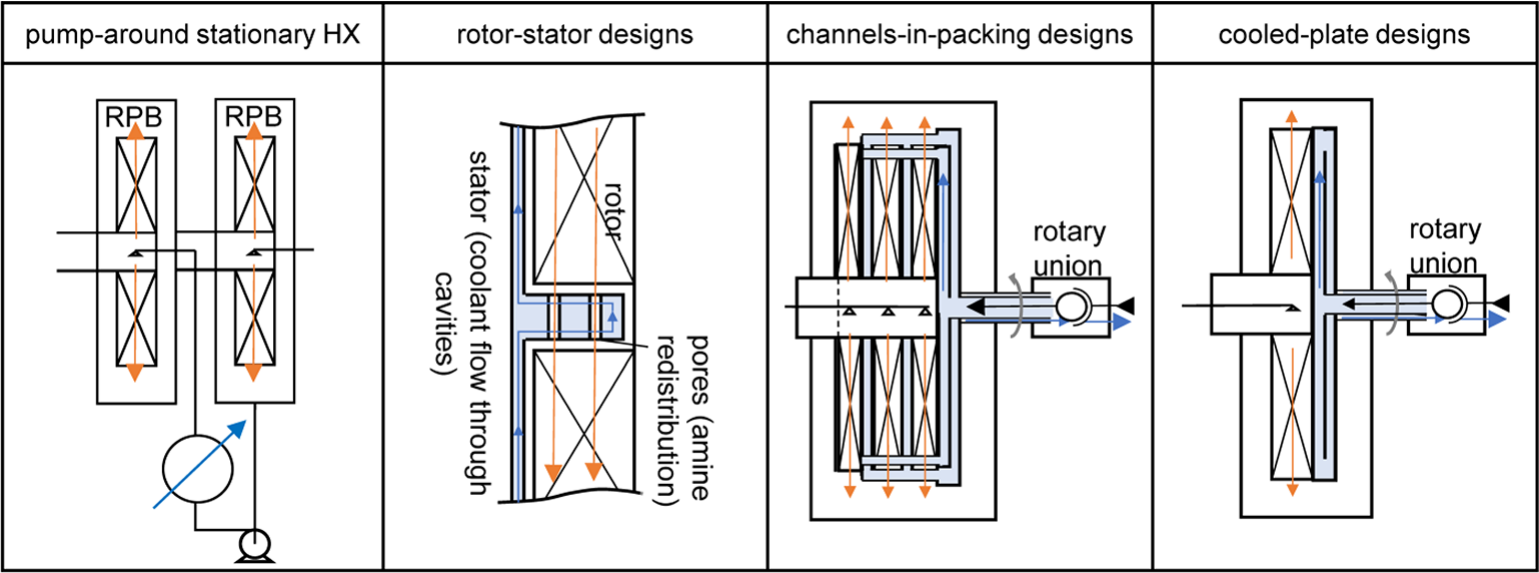
Proposed designs for intercooled RPBs from the literature.

Oko et al.^16^ identified the need for intercooling RPBs in CO_2_ capture applications. Existing designs for intercooling RPBs can be broadly grouped into four categories, depicted in Figure 1. Pump-around stationary heat exchangers use a separate heat exchanger to cool the amine between two RPB absorber stages. This is an established approach used in existing industrial pilots of RPB CCS units.^17^ It is also well established and tested in conventional columns.^1^ The disadvantage of this design is that heat can only be removed at a single point and that it requires two RPB rotor stages, increasing cost. Rotor-stator designs integrate coolant flow through cavities in the stator section of a rotor-stator RPB.^18,19^ Rotor-stator RPBs benefit from having several end-effect zones, further increasing mass-transfer coefficients.^20^ This design also avoids the need for a rotary union for coolant flow. However, higher motor power demands are required to accelerate the liquid after each stator. At full-scale, these designs need to ensure effective liquid and gas redistribution, clearance, and alignment for each stator section, increasing mechanical complexity. Channels-in-packing designs incorporate pipes, channels, or plates carrying coolant inside the RPB packing.^3,21,22^ The flexibility of this design ensures effective cooling. However, the mechanical complexity increases with scale-up. The channels also displace packing volume, eroding the intended size reduction with the potential to cause liquid distribution problems in the bed. Simpler cooled-plate designs propose to only cool the outside of the bed.^23^ This design is similar to arrangements used to cool spinning disk reactors.^24^ Larger gas throughputs in RPBs require an increased axial packing length. A cooled-plate design becomes impractical for larger RPBs as they are limited by axial conduction of heat through the packing.^25^

Review of the open literature revealed an absence of experimental results for internally cooled RPB designs including demonstration of functional prototypes and heat and mass-transfer results. In the context of carbon capture, intercooling RPBs unlock the potential of high-concentration amines. This work demonstrates a prototype design incorporating heat pipes to help alleviate the disadvantages of the previous proposed designs discussed above.

### 1.4 Heat-Pipe-Intercooled RPB for Carbon Capture

Thermosyphon heat pipes consist of sealed tubes filled with a heat-transfer fluid. The heat-transfer fluid evaporates at one end of the tube and condenses at the other, leading to very high effective thermal conductivities. As the sealed volume can be at a different pressure to atmospheric, this evaporation–condensation is not limited to the boiling point of the heat-transfer fluid and can take place across a range of temperatures.

In a chemical engineering context, heat pipes can be used for iso-thermalization (temperature flattening^26^), removing heat from chemical reactors. Commercial examples of heat-pipe heat exchangers exploit heat pipes to simplify designs in corrosive applications.^27^

In the prototype rotor described in this work, thermosyphon heat pipes are integrated into the packing of an RPB to improve the axial conduction of heat. This design is simple to scale-up as the axial length of packing can be increased to accommodate higher gas flows without significant increases in mechanical complexity. Heat pipes also separate the corrosive amine from the mild coolant-side flow. Heat-transfer performance of thermosyphon heat pipes improves under the artificial gravity of rotation^28^ according to the proportion (ω^2^*r*)^0.25^. The design reported in this work uses a rotary union, though it is possible that, in future designs, heat pipes could be used to eliminate the need for this.^29^

This work reports results from testing a novel heat-pipe-intercooled RPB rotor prototype for carbon capture in a pilot plant at Newcastle University. Results show the variable-area packing used in the design increased the upper operating limit (flooding point).^30^ Mass-transfer results demonstrate the design in a carbon-capture context and show that effective intercooling is achieved. Pressure-drop results show that the effect of liquid flow on pressure drop can be modeled through its effect on hold-up in the rotor.

## 2 Methods

### 2.1 Theory

The flooding results in this work are compared to the correlation in Lockett.^31^ This is shown in eqs 1–3 in a Sherwood flooding plot format, where *C*_g_ is the *x*-axis and *L*/*G*(ρ_g_/ρ_l_)^0.5^ is the *y*-axis. The viscosity-dependent factor (μ_l_/μ_w_)^0.03^ is unity for the water–air system.
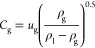
1
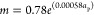
2
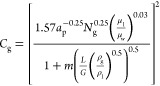
3

Mass-transfer results are presented in the form of the overall gas-side mass-transfer coefficient shown in eq 4. This is based on the number of transfer units (NTU) method and assumes that the equilibrium gas concentration of CO_2_ is negligible.^8^
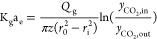
4

The pressure-drop model is given in eqs 5–8. This is based on the Ergun equation with packing voidage corrected to account for liquid hold-up using the correlation from Yang et al.^32^
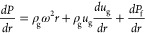
5
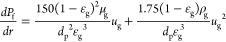
6
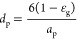
7
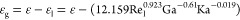
8

### 2.2 Experiment

Flooding points were determined in air–water experiments at room temperature, using the approach given in Hendry et al.^33^ The mass-transfer results from the intercooled rotor are compared to historical results from a conventional rotor reported previously^34^ for a conventional RPB rotor on the Newcastle University pilot plant. The rotors are compared in Table 1. Packings consist of an expanded metal mesh, equivalent to grades 707S (“fine expamet”) and 196S (“medium expamet”) from the supplier.^35^ Fine expamet was used for comparisons of flooding due to possessing a similar surface area and voidage to the square-mesh packing used as outer packing on the intercooled rotor. A medium experiment was used in all carbon-capture data obtained with the conventional rotor. This data set was completed as a central composite experimental design. Data points given show individual experiments at the equivalent conditions to those tested with the intercooled rotor, while the trends shown are supported by data points taken across the experimental design.

**Table 1:** Rotor Packing Properties

		inner radius	outer radius	area	voidage	axial length
rotor	packing	*r*_i_, mm	*r*_o_, mm	*a*_p_, m^2^/m^3^	ε	*z*, mm
intercooled rotor	inner packing: medium expamet (196s)	42.5	64.5	663	0.801	20
	outer packing: square mesh (1.3 mm × 1.3 mm)	65	135	1763	0.751	20
conventional rotor	medium expamet packing (196s)	42.5	140	663	0.801	20
conventional rotor	fine expamet packing (707s)	42.5	140	1492	0.788	20

**Figure 2:**
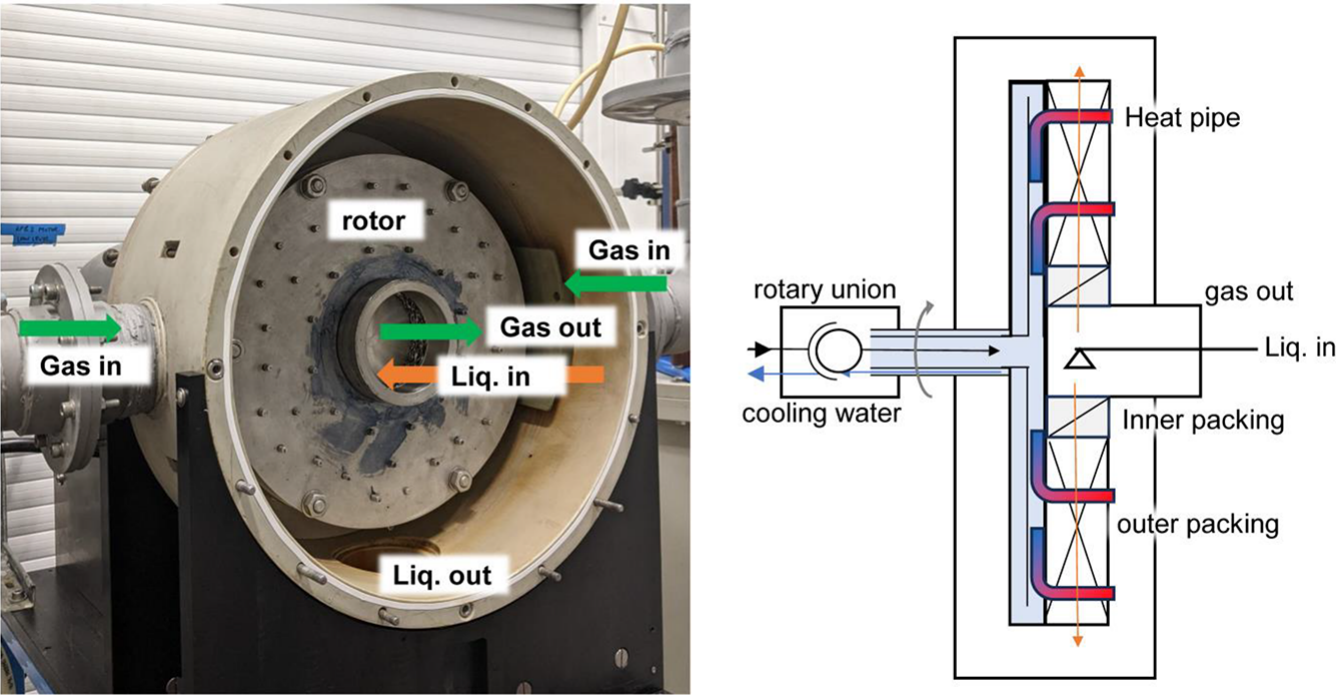
Prototype-intercooled rotor.

The prototype-intercooled rotor is shown in Figure 2. The design uses variable-area packing where the inner packing is made of more open material (higher ε, lower a_p_) than the outer packing. Variable-area packing has been shown to improve liquid hold-up in RPBs.^36^ Cooling water enters the rotor backplate via a two-way rotary union. A concentric hollow shaft and pipe distribute the cooling water into a chamber inside the backplate. The condensing end of the heat pipes is installed in the backplate and contacts the cooling water. A baffle forces the cooling water to flow over the heat-pipe condensing ends before it leaves the backplate also via a hollow shaft and two-way rotary union. The heat pipes are oriented to take advantage of centrifugal force on heat-transfer enhancement, by returning fluid to the evaporating end, installed in the packing. Forty-two heat pipes are installed at roughly equal spacing in the packing. The inner packing and outer edge of the packing do not include heat pipes because cooling the amine in these areas is not expected to make a significant contribution to performance. The heat pipes are 100 mm long and 5 mm in diameter with a 90° bend in their middle. They are made of stainless steel with a working fluid of ethanol. Spacing was designed based on a combination of expected duty in tandem with engineering CFD of the expected thermal conduction. In this experiment, a high cooling water flow rate is used to maintain an appreciable temperature difference and remove a significant amount of heat from the process. This is due to design limitations of the prototype rotor, and closer approach temperatures should be possible with future iterations of the design.

**Figure 3:**
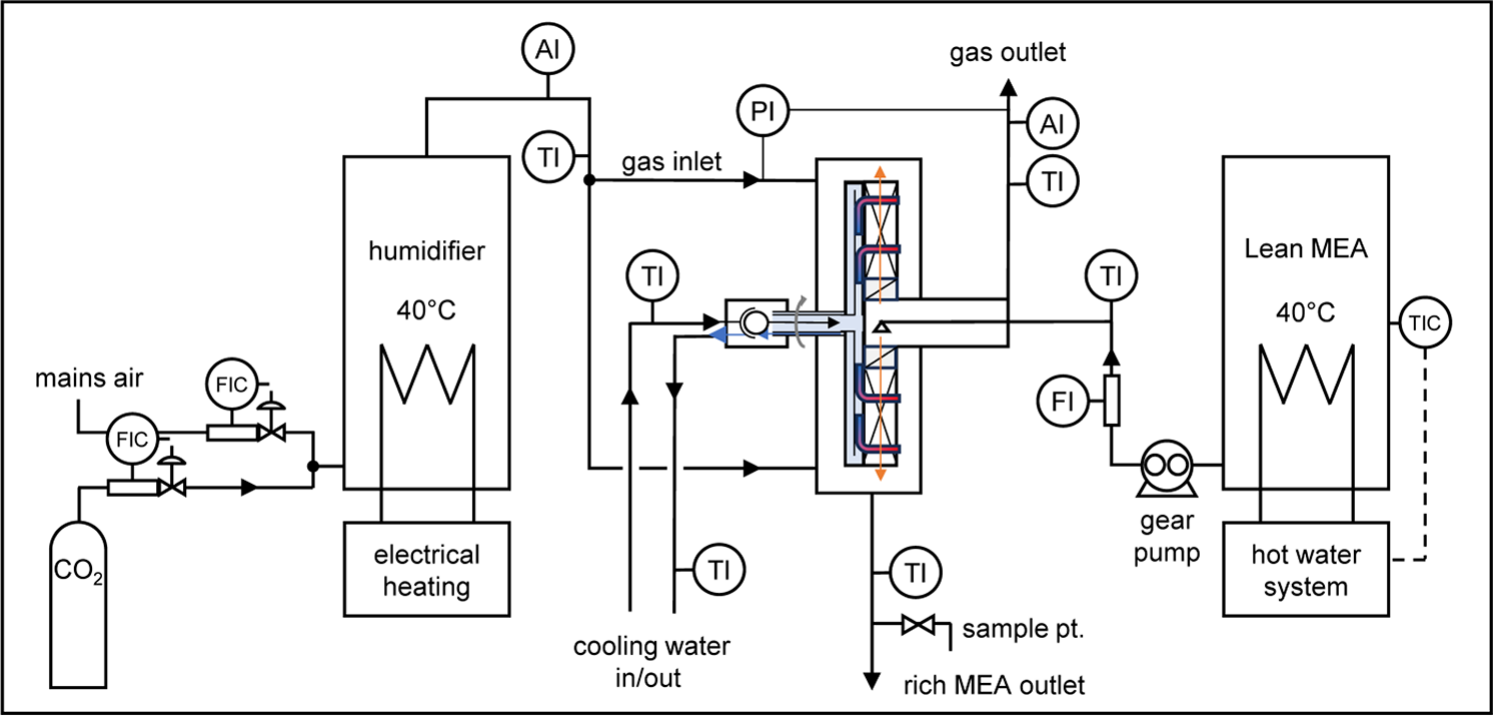
Process flow diagram of the pilot plant at Newcastle University.

The layout of the pilot plant is shown in Figure 3. Synthetic flue gas is prepared by blending CO_2_ with compressed air using gas mass flow controllers (Bronkhorst D-6383 and D-6371, FIC in Figure 3) at 12 L/s, 10 vol % CO_2_, 100%RH, and 40 °C. CO_2_ concentrations are measured at the inlet and outlet using gas analyzers (GEM Scientific Geotech G100, AI in Figure 3). Gas-side pressure drop is measured using a manometer (PI in Figure 3). Lean monoethanolamine (MEA) solution is supplied at 40 °C and at 0.15 mol of CO_2_/mol of MEA loading, monitored by a Coriolis flowmeter (Rheonik RHM 06). Temperatures (TI in Figure 3) are measured by using a combination of PT100 temperature probes and type-K thermocouples.

The amine loading and concentration were determined using a combination of the direct titration method reported in Chang^37^ together with a gasometric method using a Chittick apparatus.^38,39^ Mass balance closure was achieved to within 15%, and experimental results were repeated in duplicate.

## 3 Results and Discussion

### 3.1 Flooding Point

**Figure 4:**
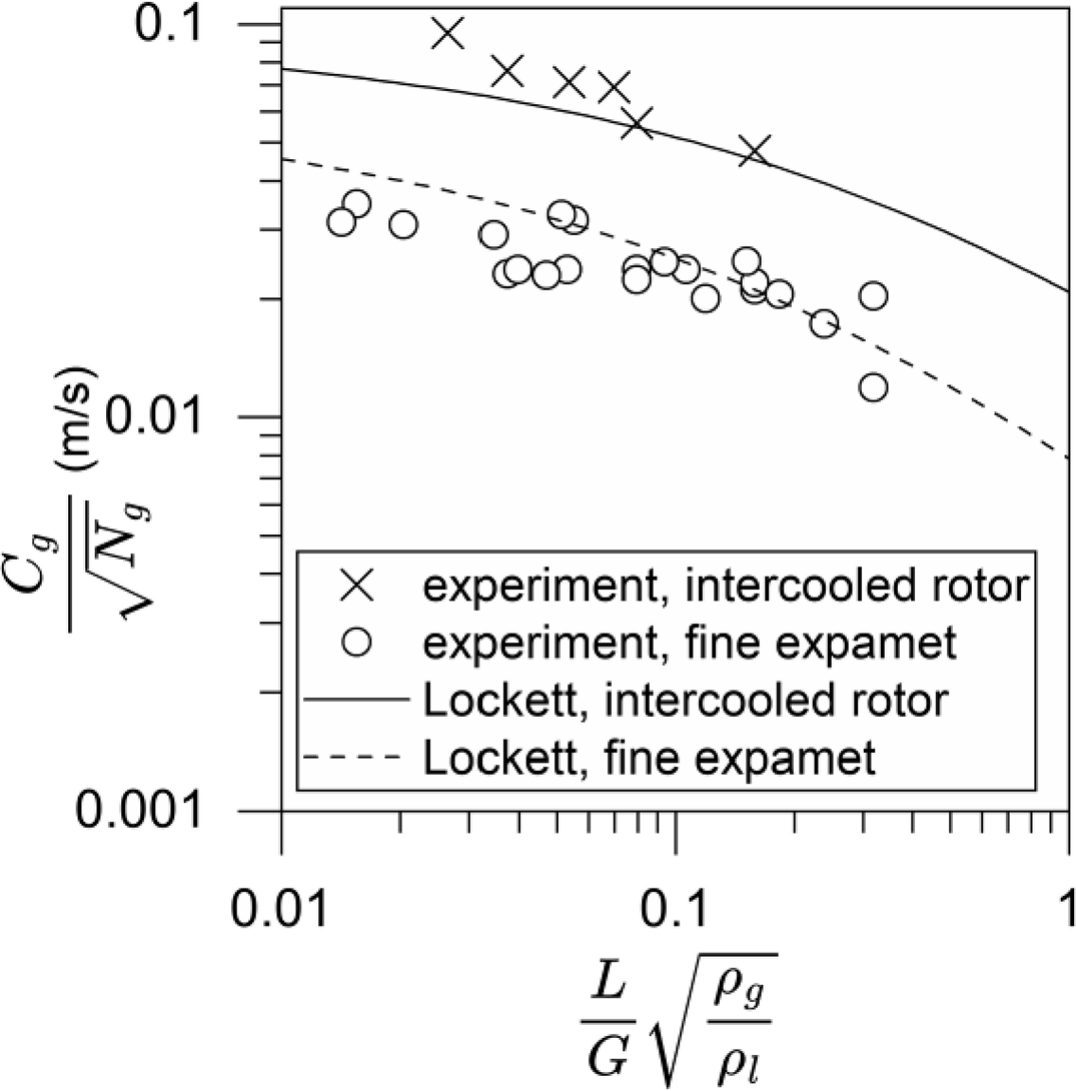
Flooding point for the intercooled RPB rotor.

Flooding points for the intercooled RPB rotor are shown in Figure 4, compared to data for a comparable high-surface-area packing (fine expamet). These experiments were completed with air and water flows only. The *X*-axis is a dimensionless flow parameter, representing the ratio of liquid-to-gas flow kinetic energies. The *Y*-axis is the gas capacity factor; this is proportional to the gas velocity and represents the kinetic energy of the gas.^30^ Division by √*N*_g_ removes the effect of rotation speed in eq 3, so that experimental data at different rotation speeds can be plotted against a single line. This plot makes the difference observable between the two rotors solely dependent on the packing properties.

The intercooled rotor is able to accommodate roughly twice the gas flow of the fine expamet rotor before flooding. This conclusion is supported by both the experimental data and fit to the Lockett correlation, which also shows a good comparison between the experiment and correlation. In practical terms, variable-area packing is capable of handling a wider range of gas and liquid flows than ordinary (uniform) packing. Variable-area packings allow the use of higher surface-area packing in the bulk of the rotor while avoiding limitations caused by flooding in the RPB eye. This results in Figure 4 serve to demonstrate this outcome experimentally. This is an important outcome for effective rotor design: for an equivalent duty, if concentrated amines reduce the rotor size by half, then the superficial gas velocity also doubles. Together with the increased viscosities, this makes flooding an important design constraint for the rotor.

### 3.2 Effect of Rotation Speed

**Figure 5:**
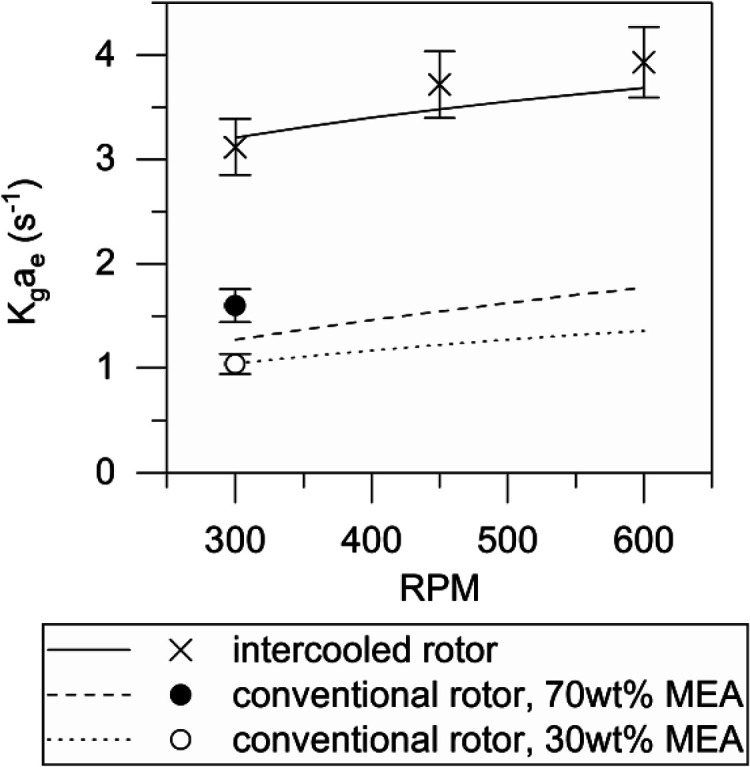
Effect of the rotation speed on mass-transfer coefficient.

Figure 5 demonstrates the influence of the rotation speed on K_g_a_e_. Experiments were completed using 70 wt % MEA at 0.15 mol of CO_2_/mol of MEA loading. The liquid flow rate was 100 kg/h at 40 °C. The gas flow rate was 12 L/s at 40 °C and 10 vol % CO_2_. The cooling water flow rate was 240 kg/h at 15 °C. The data from the intercooled rotor are compared to historical data for a conventional rotor setup at both 70 and 30 wt % amine. The experimental points shown for the conventional rotor are taken at conditions equivalent to those reported for the intercooled rotor.

Increased rotational speed improves mass transfer in RPBs by improving the wetting efficiency of packing and the rate of surface renewal. The graphs above show a similar trend for the effect of the rotation speed for the intercooled and conventional rotor. The intercooled rotor resulted in a dramatic improvement in the mass-transfer rate when compared with the conventional rotor. Compared to the conventional rotor, the mass-transfer rate has increased by 130%. From eq 4, K_g_a_e_ is inversely proportional to the rotor volume, demonstrating that a halving in equipment size is achieved using the intercooled rotor. By comparison, conventional columns equipped with modern structured packings,^40^ operating with 30 wt % MEA at pilot-scale deliver K_g_a_e_ ≈ 0.18 s^–1^, suggesting a 20× volume reduction compared to conventional technology. Comparison at the two concentrations also indicates that this performance increase is not explained solely by the increase in the amine concentration from 30 to 70 wt %. The performance increase is due to internal cooling and packing changes implemented on the intercooled rotor.

### 3.3 Effect of the Liquid Flow Rate

**Figure 6:**
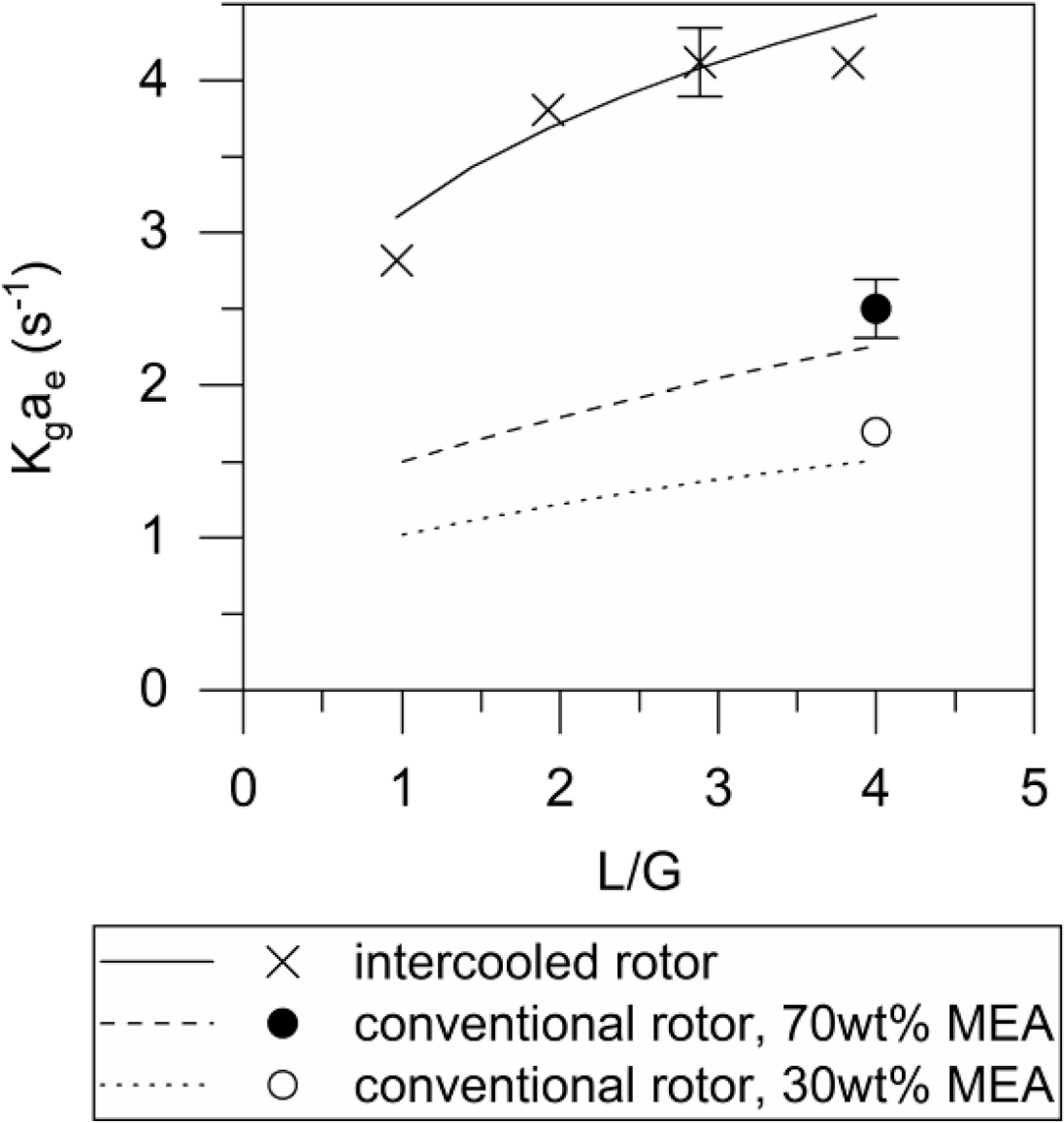
Effect of the liquid flow rate on mass-transfer coefficient.

The effect of the liquid flow rate on mass transfer is presented in Figure 6. These experiments used 70 wt % MEA at 0.15 mol CO_2_/mol MEA loading and 40 °C. The gas flow rate was constant at 12 L/s, 40 °C, and 10 vol % CO_2_. The cooling water flow rate was 240 kg/h at 15 °C. The results are presented in terms of the *L*/*G* ratio. *L*/*G* dictates the rich amine loading required for efficient regeneration and therefore the efficiency of the absorption process. Concentrated amines can reduce equipment size for the whole CCS plant, by reducing the solvent flow rate, which decreases *L*/*G*. There may be a risk of incomplete wetting to consider for intercooled RPB designs. These results show that the performance improvement achieved with the intercooled rotor is maintained at a lower *L*/*G*.

Inside the variable-area packing rotor, the abrupt increase in packing surface area between the inner and outer packing creates an abrupt increase in liquid hold-up. Previous workers^36^ have demonstrated this experimentally. The mass transfer has been investigated previously for the CO_2_–NaOH_(aq)_ system.^41^ The result in Figures 5 and 6 provides an experimental demonstration of variable-area packing in a pilot-scale CCS context using a MEA solvent.

The K_g_a_e_ obtained experimentally was correlated against the rotation (gravitational force equivalent) and the liquid flow (liquid Reynolds number) using the average radius 88.75 mm as a reference. The resulting correlation is shown in eq 9. This was used to generate lines for the intercooled rotor in Figures 5 and 6. The coefficient of determination *R*^2^ = 0.81. The impact of both liquid flow and rotation demonstrate performance plateaus as both are increased: as rotation removes resistances to mass transfer, performance will become kinetically limited. As the liquid flow rate increases, complete wetting of the packing is achieved, and beyond this point, there are not further significant benefits.
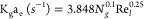
9

### 3.4 Cooling Duty

**Figure 7:**
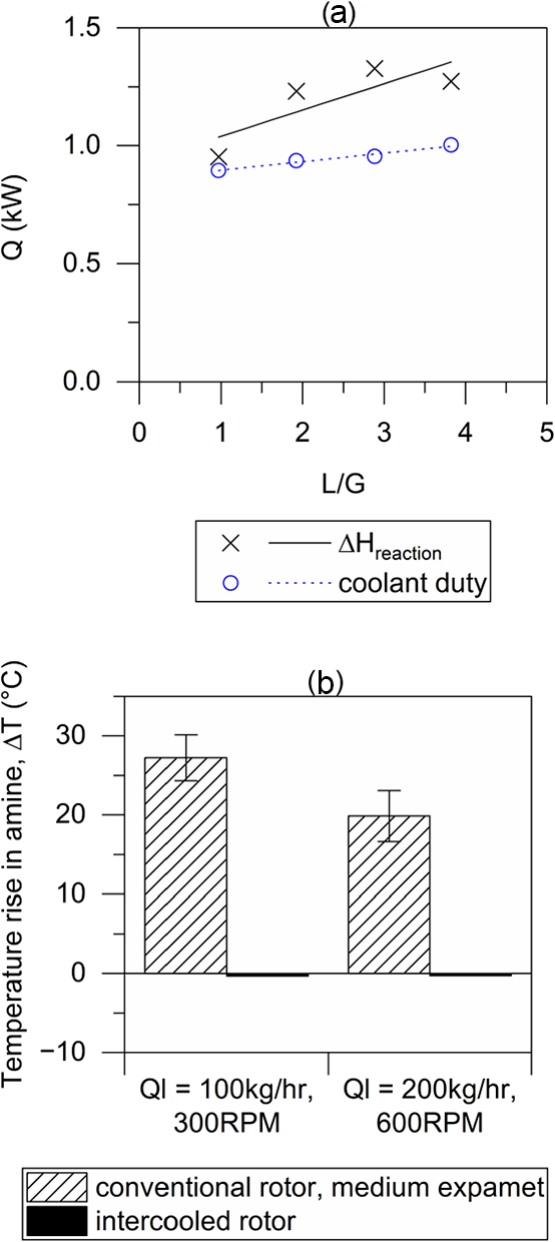
Coolant duty for the intercooled rotor. (a) Comparison of heat-of-reaction with coolant duty. (b) Comparison of a temperature rise in outlet amine against data for a conventional rotor.

The coolant duty for the intercooled rotor is illustrated in Figure 7a under the same experimental conditions as those reported in Figure 6 above. The heat-of-reaction based on the CO_2_ absorption rate is met by the cooling duty of the rotor based on temperature and the flow rate of the cooling water. The remaining energy difference is of the order of 200W and can be explained by losses to the environment. During the campaign, virtually no temperature increase in the outlet amine was observed with the intercooled rotor. In comparison, without intercooling, the conventional rotor showed a temperature rise for the outlet amine of 15–30 °C: a comparison is shown in Figure 7b for the two conditions tested. This shows that effective intercooling is achieved in this rotor design. This also suggests it may be possible to achieve isothermal conditions during CCS absorption in future using intercooled RPBs.

### 3.5 Pressure Drop

**Figure 8:**
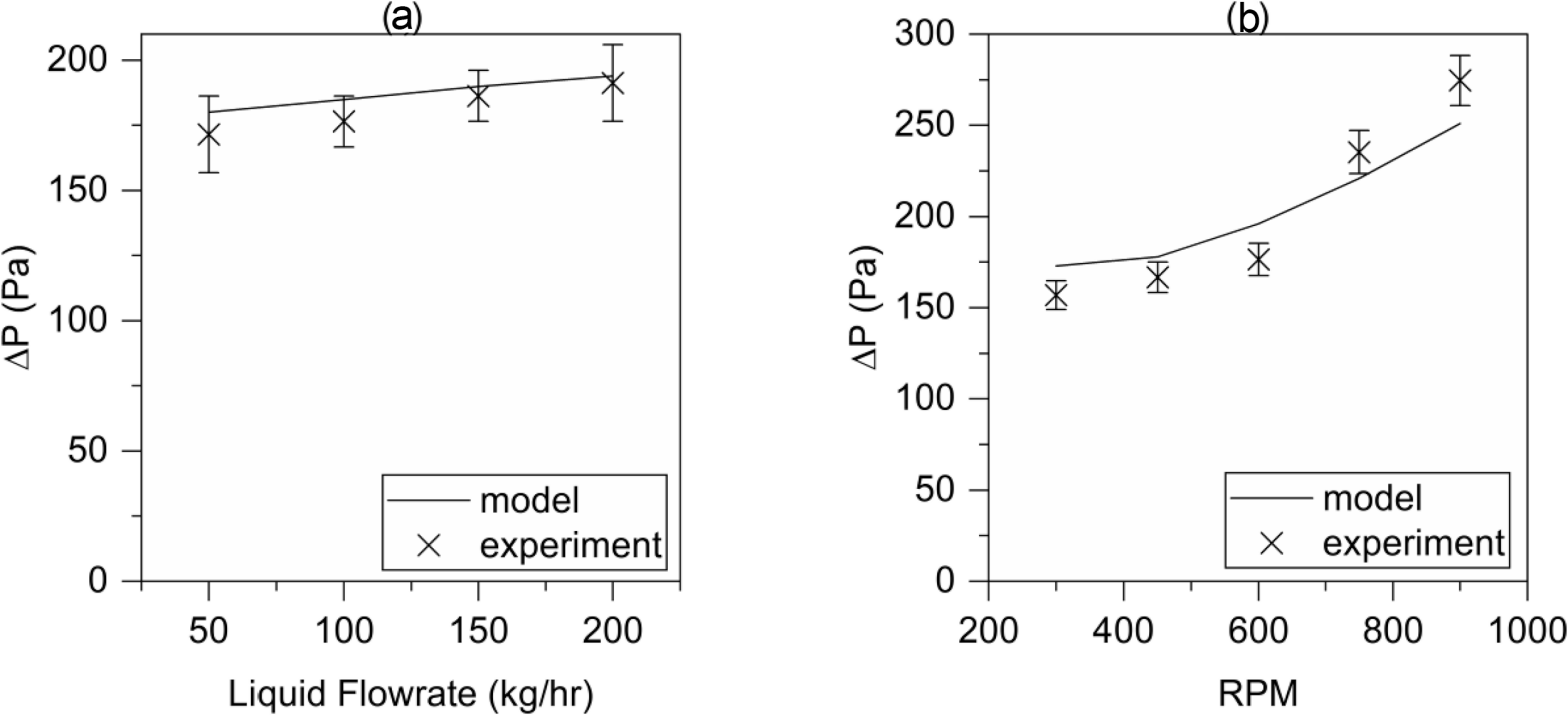
Pressure-drop experimental data vs model. (a) Effect of the liquid flow rate and (b) effect of rotation speed.

The effects of the liquid flow rate and rotation speed on pressure drop are given in Figure 8. For pressure-drop measurements completed during amine capture testing (Figures 5 and 6), the model described in eqs 5–8 provides a good explanation for the effect of liquid flow in Figure 8a. The effect of RPM is given in Figure 8b. The main upward trend in these data is explained by solid-body rotation of the gas within the rotor. At lower speeds, there is an increased liquid hold-up in the packing. As the voids fill with liquid, this increases the pressure drop compensating for the reduced rotation speed. At 900 rpm, the pressure drop exceeds the predictions. This trend, however, could be explained by solid-body rotation in the area immediately beyond the packing’s outer radius.

## 4 Conclusions

This work presents results from pilot-plant testing of a novel heat-pipe-intercooled rotating packed bed rotor. The results demonstrate that a further halving in equipment size vs conventional rotor assemblies is possible using the intercooled design. This is demonstrated across rotation speeds and *L*/*G* ratios, representative of operating conditions in a full-scale RPB CCS process. Heat-transfer results during the experiment show that a majority of the heat is successfully removed during intercooling. The effect of the liquid flow rate on pressure drop can be estimated by a simple model that accounts for the effect of liquid filling voids in the packing. In keeping with a process intensification philosophy, heat and mass-transfer processes are combined in a single device, achieved using heat-pipe integration, and under rotation in the RPB, solvent concentrations are increased substantially. These factors work together in a mutually beneficial way to deliver further process improvement of RPB CCS processes.

## Supporting Information Available

Table of raw historical data for the conventional rotor (PDF)

## Abbreviations

CAPEX: Capital expenditure
CCS: carbon capture and sequestration
CFD: computational fluid dynamics
HX: heat exchanger
MEA: monoethanolamine
NTU: number of transfer units
RH: relative humidity
RPB: rotating packed bed.
**Nomenclature Table**
*a*_p_: packing surface area per unit bed volume m^2^/m^3^
*C*_g_: gas capacity factor m/s
*d*_p_: effective particle diameter (packing) m
Galileo number
Kapitza number
K_g_a_e_: gas-side overall mass-transfer coefficient s^–1^
*L*/*G*: liquid–gas flow ratio, mass basis (kg/s per kg/s)
*m*: empirical constant in flooding correlation
G-number: gravitational force equivalent.
NTU: number of transfer units (overall gas-side)
*P*: pressure Pa
*Q*: heat flow kW
*Q*_g_: gas flow rate m^3^/s
*Q*_l_: liquid flow rate kg/h
*r*: bed radius (*r*_I_ = inner bed radius, *r*_o_ = outer bed radius) m
Reynolds number for the liquid (u_l_ρ_l_ = liquid’s superficial mass velocity, radial, relative to packing (kg/m^2^s))
*u*_g_: superficial gas velocity (radial, relative to packing) m/s
*z*: axial length of the bed m
Δ*H*_reaction_: heat released during the absorption kW
α: loading. dissolved CO_2_ in amine (mol_CO2_/mol_amine_)
ε: packing voidage per unit bed volume (m^3^/m^3^ bed)
ε_g_: gas hold-up per unit bed volume (m^3^/m^3^ bed)
ε_l_: liquid hold-up per unit bed volume (m^3^/m^3^ bed)
μ_l_: liquid viscosity Pa.s
μ_g_: gas viscosity Pa.s
ρ_g_: gas density kg/m^3^
ρ_l_: liquid density kg/m^3^
σ: liquid surface tension Nm
ω: angular velocity rad/s
