# Supplementary material for: Pilot-Scale Carbon Capture in a Heat-Pipe-Intercooled Rotating Packed Bed
Source: Ind Eng Chem Res. 2025 Jan 23;64(5):2872–9. doi: 10.1021/acs.iecr.4c01614 (PMC11803622; doi:10.1021/acs.iecr.4c01614)
Supplement: Supplementary file 1 — ie4c01614_si_001.pdf [file ie4c01614_si_001.pdf]

Supporting Information

Pilot-Scale Carbon-Capture in a Heat-Pipe Intercooled Rotating Packed Bed

James R. Hendry, Jonathan G.M. Lee*

School of Engineering, Merz Court, Newcastle University, Newcastle upon Tyne, NE1 7RU, UK.

Corresponding Author

*James Hendry. james.hendry@newcastle.ac.uk. School of Engineering, Merz Court, Newcastle University, Newcastle upon Tyne, NE1 7RU, UK. +44(0)191 208 5747.

This paper compares the intercooled rotor performance against historical data for amine absorption using a conventional rotor with medium expamet. This data has formed part of a previous publication, in Luo et al[32].

(32) Luo, X.; Wang, M.; Lee, J.; Hendry, J. Dynamic modelling based on surface renewal theory, model validation and process analysis of rotating packed bed absorber for carbon capture. *Applied Energy* 2021, *301*, 117462. DOI: https://doi.org/10.1016/j.apenergy.2021.117462.

The data is reproduced below. The experiments follow a central composite experimental design. A response surface model was used to present the data graphically, this has also been included below.

Table S1. Historical data, conventional rotor, expamet medium, counter-current.

			gas analysers:		titrations: inlet		outlet		temperatures				$K_g a_e$
Data pt.		Q_l	y_{in} CO_2	y_{out} CO_2	inlet amine	inlet α	sump α	rotor α	Liquid Out	Liquid In	Liquid-Sump	Air in	gas-side
	RPM	(kg/hr)	(vol.fr.)	(vol.fr.)	(wt%)	(mol%)	(mol%)	(mol%)	(°C)	(°C)	(°C)	(°C)	(s^{-1})
1	300	105.5	0.1175	0.107	33.7%	20.8%	25.2%	23.7%	68	32	68	36	0.99
1	300	104.3	0.124	0.112	33.7%	20.8%	25.3%	24.4%	57	37	55	32	1.08
2	1000	106.2	0.121	0.105	33.7%	20.8%	25.4%	24.6%	64	32	64	36	1.50
2	1000	104.3	0.1255	0.108	33.7%	20.8%	26.7%	26.2%	56	39	56	33	1.59
3	300	317.2	0.125	0.107	33.7%	20.8%	23.0%	22.0%	52	33	47	34	1.62
3	300	317	0.117	0.101	33.3%	22.8%	25.5%	24.8%	45	42	45	30	1.51
4	1000	318.1	0.124	0.1015	33.7%	20.8%	23.6%	22.7%	53	34	47	35	2.12
4	1000	317.2	0.123	0.105	33.3%	22.8%	24.6%	23.9%	40	27	35	31	1.69
5	300	105.3	0.1030	0.0880	62.0%	18.7%	21.5%	20.6%	65	41	64	35	1.67
5	300	106	0.101	0.0875	60.9%	20.3%	22.6%	21.8%	68	37	68	29	1.52
6	1000	105.3	0.1000	0.0810	62.0%	18.7%	22.7%	21.9%	69	40	70	36	2.24
6	1000	105.2	0.1030	0.0810	62.0%	18.7%	22.1%	21.9%	65	40	66	35	2.55
6	1000	106.1	0.103	0.0835	60.9%	20.3%	24.3%	23.3%	71	37	71	32	2.23
7	300	317.9	0.1035	0.0835	60.9%	20.3%	21.6%	20.7%	61	40	52	29	2.28
9	300	213.1	0.104	0.087	42.4%	18.4%	20.1%	19.4%	53	34	49	31	1.89
9	300	211.2	0.110	0.090	42.4%	18.4%	20.8%	20.1%	53	39	50	31	2.12
10	1000	211.9	0.107	0.084	41.7%	19.6%	22.8%	22.2%	60	41	58	36	2.57
11	650	105.5	0.110	0.090	42.4%	18.4%	23.6%	22.9%	54	40	54	31	2.13
11	650	105.7	0.109	0.090	41.7%	19.6%	24.7%	23.1%	61	41	60	36	2.03
11	650	105.6	0.1085	0.090	41.7%	19.6%	25.3%	23.6%	56	41	56	34	2.04
11	650	105.6	0.109	0.091	41.7%	19.6%	24.0%	23.9%	52	41	53	32	1.97
12	650	319.1	0.109	0.084	42.4%	18.4%	20.3%	20.1%	52	38	47	31	2.72
12	650	317.7	0.1095	0.086	41.7%	19.6%	22.3%	22.0%	52	41	49	33	2.63
12	650	318.9	0.1	0.079	41.7%	19.6%	22.3%	21.9%	49	41	47	32	2.55
13	650	211.2	0.124	0.104	33.7%	20.8%	23.9%	23.2%	55	37	52	34	1.92
13	650	211.5	0.1255	0.105	33.7%	20.8%	25.4%	24.1%	52	42	51	33	1.94
13	650	209.1	0.114	0.102	33.3%	22.8%	26.5%	25.4%	46	36	43	30	1.23
13	650	210.7	0.114	0.098	33.3%	22.8%	26.3%	25.4%	48	42	47	30	1.60
14	650	212.3	0.1025	0.08	60.9%	20.3%	22.9%	21.2%	64	39	60	30	2.63
14	650	211.5	0.104	0.0825	60.9%	20.3%	21.8%	21.5%	60	41	57	29	2.46
15	650	211.6	0.108	0.085	42.4%	18.4%	21.1%	19.8%	51	41	50	31	2.60
15	650	201.9	0.109	0.087	41.7%	19.6%	23.2%	22.2%	57	41	55	35	2.39
15	650	211	0.1075	0.086	41.7%	19.6%	22.7%	22.5%	50	41	49	32	2.33

Response Surface Model for K_gA (s^{-1}) for expamet medium, counter-current

$$K_gA = -3.493 + 0.0857A + 0.1971B + 16.77C - 0.003646A^2 - 0.01871B^2 - 17.46C^2 - 0.00132AB + 0.1081AC + 0.2446BC$$

Where:

A = rotation speed, in Hz

B = L/G mass flow ratio

C = MEA concentration, wt% as a fraction.

Example calculation:

For experimental conditions of 1000 RPM, L/G = 6, 32wt% MEA:

A = 1000/60 = 16.67 Hz

B = 6

C = 0.32

$$K_gA = -3.493 + 0.0857 * 16.67 + 0.1971 * 6 + 16.77 * 0.32 - 0.003646 * 16.67^2 - 0.01871 * 6^2 - 17.46 * 0.32^2 - 0.00132 * 16.67 * 6 + 0.1081 * 16.67 * 0.32 + 0.2446 * 6 * 0.32$$

$K_gA = 1.92\ s^{-1}$
